# Ear Prosthesis for Postburn Deformity

**DOI:** 10.1155/2018/2689098

**Published:** 2018-04-29

**Authors:** Alagesan Chinnasamy, Vidhya Gopinath, Ashish R. Jain

**Affiliations:** ^1^Melbourne Dental School, The University of Melbourne, Melbourne, VIC, Australia; ^2^Department of Prosthodontics, Saveetha Dental College and Hospitals, Saveetha University, Chennai, India

## Abstract

Prosthodontics is not just confined to replacement of missing teeth but beyond one's scope. The fabrication of any extraoral maxillofacial prosthesis presents the prosthodontist with several phenomenal challenges. Psychologically, these patients are severely affected either by congenital absence or loss of ear due to trauma or burns. Replacement or reconstruction can be done by surgical or prosthetic approach. However, not all situations are favourable to surgical reconstruction. This article emphasises on the steps in fabrication of ear prosthesis for burn deformity.

## 1. Introduction

Loss of external ear can be congenital or acquired due to accidental trauma or malignant disease. Congenital anomaly of the external ear may be termed as “microtia.” It includes a spectrum of deformities from a grossly normal but small ear to the absence of the entire external ear. These deformities account for three in every 10,000 births, with bilaterally missing ears seen in fewer than 10% of all cases [1–3]. The patient presented in this article had a unilateral acquired missing ear due to burn deformity. Leading life with this kind of physical deformity is very stressful and often depressing for the patient. It directly affects the patient's mental, social, and psychological well-being. Acceptable aesthetics in restoring a prominent facial defect, such as malformed ear, is a challenging task for a maxillofacial prosthodontist. Maxillofacial training is an essential part of postgraduate curriculum in prosthodontics. Oral malignancy, facial tumours, trauma, and burns most of the time require rehabilitation either by surgery or prosthetic approach [4–8]. Poly(methyl methacrylate), poly(vinyl chloride), polyurethane, and silicone are the materials of choice for prosthetic rehabilitation as they are biologically accepted. However, silicones are preferred due to its flexibility, light weight, and lifelike appearance. A surgical approach may not be a treatment of choice most of the time due to certain constrains. In the face of these deformities and limitations, the surgical goals of reconstruction vary and can be very challenging, and this article presents a simple procedure for reconstruction of missing ear due to burns. A prosthetic ear is an artificial ear that is usually made of silicone from a mould that is prepared using the opposite ear (or a parent's ear in bilateral cases) as a template [9–13]. The prosthesis can either be held onto the head with adhesive or using magnets or clips (requires minor surgical procedure) or can be attached to the spectacle. The function of the prosthetic ear shape is not just confined to cosmetic reconstruction but it also directs the sound waves into the auditory canal and to maintain a proper environment for the inner ear membranes. It normally improves hearing by about 20%. The prosthetic ear will retain eyeglasses and retain a hearing aid if needed. It also serves as a great psychological benefit in the rehabilitation of the patient's social, physical, and mental well-being [14, 15].

## 2. Case Report

**Figure 1:**
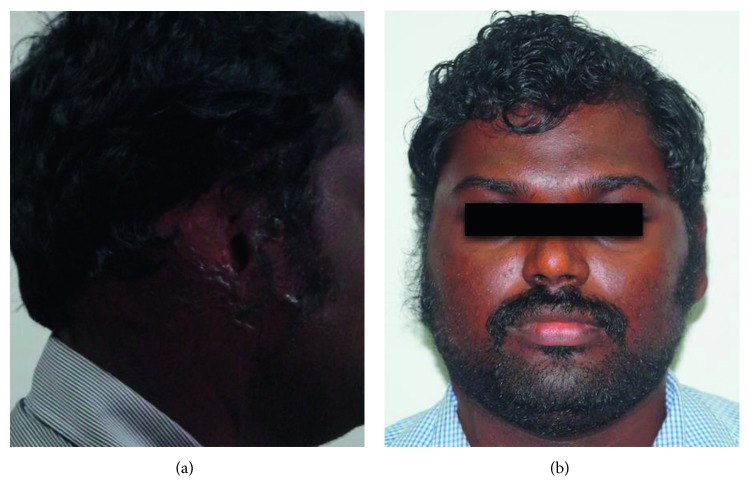
Profile and frontal photographs of patient missing ear.

A 28-year-old male patient who lost his right ear in an electric burn reported to the Department of Prosthodontics, Saveetha Dental College and Hospital, for further treatment. Examination and history revealed that the patient had suffered from electric burns with scar formation, but some rudimentary ear was present in the tragus region of missing right ear (Figure 1). Hearing capability was not compromised on both sides. A prosthetic reconstruction was decided to fulfil the patient's desire of cosmetic correction without surgery.

### 2.1. Impression Procedures

**Figure 2:**
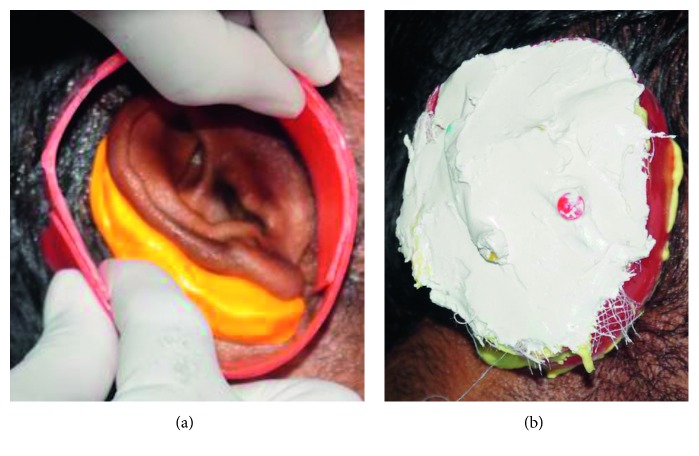
Impression of donor ear using irreversible hydrocolloid.

**Figure 3:**
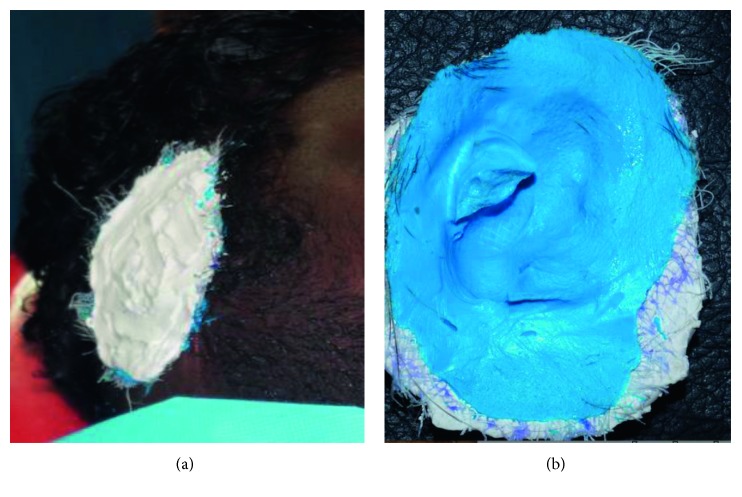
Impression of missing ear using polyvinylsiloxane.

Examination of the defect showed healthy tissue with scar formation and rudimentary tissue tag at the height of the tragus, and the contralateral ear was normal. Before the impression making, three horizontal markings are made on the normal ear at the junction of helix with the side of the head as the first marking, the second at the middle of the tragus, and the last one at the junction of the ear lobe with the side of the head. Similar markings were made on the defective side, so that these markings were accurately transferred to the working cast. The borders were confined and closed by hair and smeared with vaseline. While making an impression, the external auditory canal was blocked with a cotton plug and the impression was made by irreversible hydrocolloid and with the gauge in between, and backing is done by quick-setting plaster to provide support for the impression (Figure 2). The impression of the donor ear was made with light body additional silicone, and the impression was poured with the dental stone to get the master cast of the defect site and the cast of the normal ear (Figure 3).

### 2.2. Sculpting

**Figure 4:**
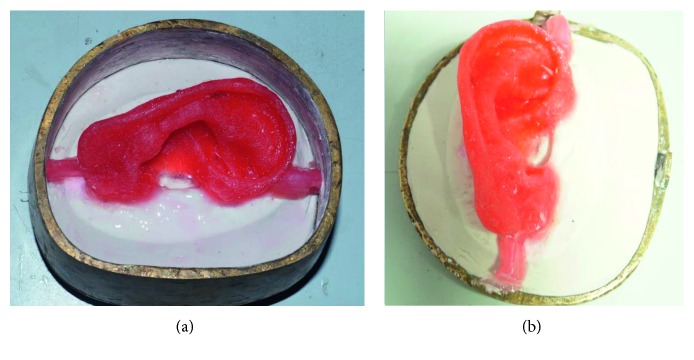
Fabrication using three piece mould system.

The prosthesis should have a lifelike appearance, so sculpting was done with utmost care to complement the aesthetics. The prosthesis can be sculpted from the beginning or the donor technique may be used. The donor ear can be selected from a sibling or from any person whose ear matches with the patient's ear. For this patient, the donor technique was used and the impression was made and filled with modelling wax and later retrieved from the impression (Figure 4(a)).

### 2.3. Surface Die Fabrication: Three Piece Die

After the wax pattern try-in was done, it was oriented on the master cast using the markings placed on the defect site and necessary modification was done to match the contralateral ear. The wax pattern was then sealed in position on the master cast and leading edges were thinned as much as possible so as to allow the silicone edges to feather into the natural skin and when used in conjunction with adhesive they disappear. A three piece mould was fabricated for easy placement of silicone in the mould. To get the three piece mould, top portion of the dental flask was used as the base, the master cast along with the wax ear pattern was placed on this top portion of the flask, and dental plaster was poured to flush with the surface of the cast leaving no undercuts (Figure 4). Once set, two grooves were created on the plaster at the back portion of the ear to reorient the piece of mould. Separating media was applied as a separating agent and the dental stone was mixed and filled on the back side of the ear wax pattern to flush just underneath the superior margin of the helix extending till the base of the helix and junction of the lobe with the side of the head without leaving any undercut. Once the second pour was set, similar grooving procedure was carried out and the third pour was done using plaster, and the lid was placed and clamped and allowed to set.

### 2.4. Packing

**Figure 5:**
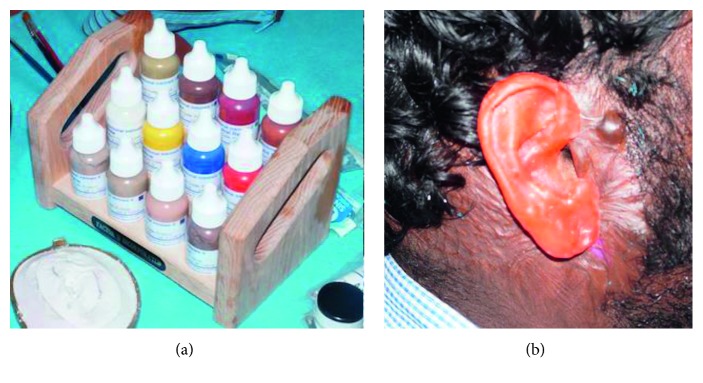
Colour matching using intrinsic factor II stain.

**Figure 6:**
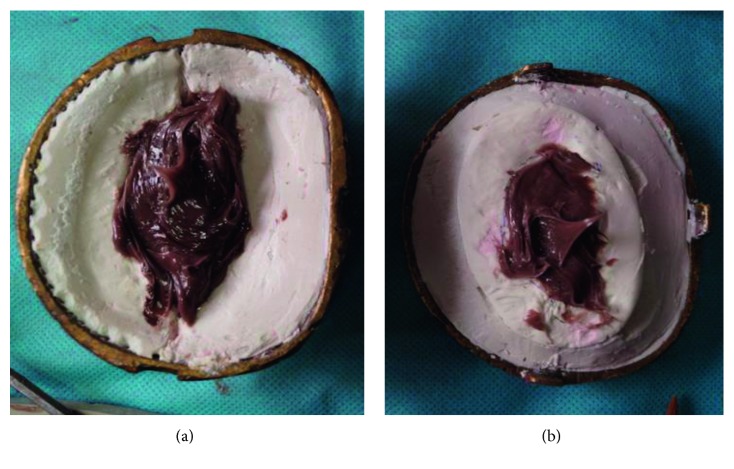
Packing using dark and light shades of RTV silicones.

**Figure 7:**
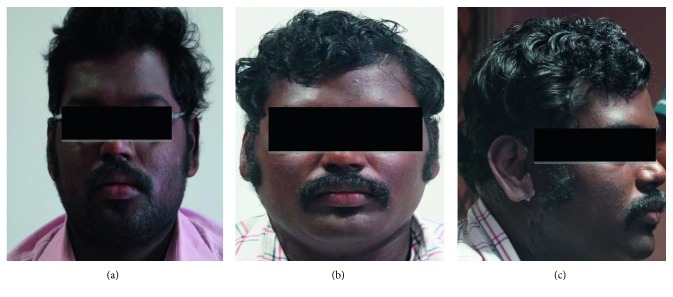
Frontal and profile photographs after insertion of ear prosthesis.

Dewaxing was done in the usual manner. After keeping the flask in hot water for fifteen minutes, the flask was opened carefully and all three pieces of mould were thoroughly cleaned with hot water to remove traces of petroleum jelly and wax. Cold mould seal was diluted with water in 1 : 1 ratio and applied. The moulds were allowed to dry completely as traces of vaseline, wax, or water will interfere with setting of silicone and the prosthesis will have the tacky surface that will invite catching of dust at a later date. The three piece mould was now ready for silicone packing. Medical-grade factor-II room temperature vulcanising (RTV) silicone was used and was mixed as per the manufacturer's instruction. Thixo was added to prevent porosity in the prosthesis. To fabricate a lifelike silicone prosthesis, the patient must always be present for the colour match. It requires great care and patience from the doctor along with an understanding of colour matching for a successful result. As the silicone is translucent, desired skin colour can be obtained using primary colours in proper proportions. Using red, yellow, and blue primary colours of intrinsic colouring system, the first base shade was prepared, and then as per the requirement, additional shades were prepared. Care was taken while adding colour, as colour loading will lead to opacity and lifeless appearance (Figure 5). To match with the patient's normal ear, the dark and light shade silicone was poured in the mould. To create the characteristics, red flocks were placed on the surface layer and the mould was packed and allowed for bench curing for 24 hours. Cured prosthesis was retrieved from the mould, cleaned thoroughly with soap, and the excess silicone flesh was trimmed from the margins (Figure 6). The prosthesis was tried on the patient and margins were trimmed as per requirement, and then the prosthesis was attached to the spectacle frame and placed in position (Figure 7).

### 2.5. Retention and Care

An instruction leaflet with “Do's and Dont's” explained in it was given to the patient with an instruction that the prosthetic ear should be replaced every few years as the old one wears off. The skin surface should be maintained clean.

## 3. Discussion

The choice between surgical reconstruction and prosthetic restoration of facial defects is a difficult decision. As consistent good results have not been demonstrated in staged surgical ear reconstruction, the prosthetic restoration is the preferred option. There are various techniques for fabrication of ear prosthesis: conventional technique, shaper/tracer technique, photocopying technique, computerised tomography (CT) scanning, magnetic resonance imaging (MRI), 3-D laser scanning, computer numerically controlled (CNC) milling, rapid prototyping, and stereolithography [4–9]. The prosthesis made by CAD/CAM techniques is better than that fabricated by conventional methods. Unfortunately, taking into account the complexity and the high cost of the equipment needed, these techniques can only be used in well-developed establishments and an academic institution which makes us rely on more conventional techniques for the fabrication of extraoral maxillofacial prosthesis. The application of osseointegrated ear implants has changed the patient perception about facial prosthesis because of effective retention and improved aesthetics. However, it requires sufficient healthy bone at the defect site for implant placement, surgical intervention, cost involvement, and usually a time interval between implant placement and prosthetic rehabilitation. On the contrary, adhesive-retained prosthesis can be placed immediately on a healthy tissue bed, without surgery and is cost-effective [11–13]. Many techniques are in use for fabrication of wax pattern for adhesive-retained silicone ear prosthesis. One among them is sculpting the pattern by carving the wax. The second is a slicing technique in which the wax pattern is made using slices of wax pattern of normal ear and placing them in opposite directions [14, 15]. The technique used in this methodology was modification of donor ear which allows easy, quick incorporation of finer anatomical details making it more lifelike appearance. With the advancement in technologies, CAD/CAM is also being used for scanning and three dimensional reconstruction of ear, but it requires special armamentarium which may not be freely accessible and not cost-effective.

## 4. Conclusion

This article presents an outline of the fabrication procedure in constructing an ear prosthesis with three piece stone mould for processing silicone. Medical-grade silicone maxillofacial prosthetic material has the ability to match any skin colour using intrinsic and extrinsic colour system and is colour stable, has rubbery consistency to match the elasticity of the skin, is biologically inert, and is thus biocompatible. An aesthetic spectacle retained ear prosthesis was made by a meticulous step-by-step procedure using donor ear for fabrication of wax pattern. Rehabilitation of a patient with missing ear due to any condition can be achieved by restoring the defect and fulfilling the objective of maintaining patient comfort, aesthetics, and bringing the patient back to the society to lead a normal life.
